# Preliminary study of the Southampton Hand Assessment Procedure for Children and its reliability

**DOI:** 10.1186/1471-2474-15-199

**Published:** 2014-06-10

**Authors:** Ecaterina Vasluian, Raoul M Bongers, Heleen A Reinders-Messelink, Pieter U Dijkstra, Corry K van der Sluis

**Affiliations:** 1 Department of Rehabilitation Medicine, University of Groningen, University Medical Center Groningen, Groningen, The Netherlands; 2 University of Groningen, University Medical Center Groningen, Center of Human Movement Sciences, Groningen, The Netherlands; 3 Department of Oral and Maxillofacial Surgery, University of Groningen, University Medical Center Groningen, Groningen, The Netherlands; 4 Rehabilitation Center ‘Revalidatie Friesland’, Beetsterzwaag, The Netherlands

**Keywords:** Reproducibility of results, Intra-observer variability, Inter-observer variability, Treatment outcome, Disability evaluation, Hand injuries, Physical and Rehabilitation Medicine

## Abstract

**Background:**

The Southampton Hand Assessment Procedure (SHAP) is currently used in the adult population for evaluating the functionality of impaired or prosthetic hands. The SHAP cannot be used for children because of the relatively larger size of the objects used to perform SHAP tasks and unknown clinimetric properties. The aims of this study were to adapt the SHAP for use in children (SHAP-C), to determine norm values for the SHAP-C, and to analyze the reliability of the SHAP-C.

**Methods:**

The SHAP-C was adapted based on the SHAP protocol. Some objects were downsized, and the timing of tasks was performed by the rater instead of the participant. Intra- and inter-rater reliability were assessed in 24 children (5 [0.54] y/o) with unimpaired hands. The repeatability coefficients (RCs) were calculated. An RC ≤ 75% of the mean SHAP-C task values was considered good reliability.

**Results:**

Participants were able to perform all SHAP-C tasks. The means of the SHAP-C tasks ranged from 0.75 to 1.21 seconds for abstract objects and from 0.64-19.13 seconds for activities of daily living. The RCs of a single assessor did not exceed 75% in 17/26 SHAP-C tasks, displaying a relatively good intra-rater reliability, whereas the RCs for the inter-rater reliability exceeded 75% in 22/26 SHAP-C tasks, thus displaying poor reliability.

**Conclusion:**

In this first study that adjusted the SHAP for pediatric use, we found that all SHAP-C objects and tasks could be performed by children. The intra-rater reliability was better than the inter-rater reliability. Although the SHAP-C appears to be a promising instrument, the protocol requires further modifications to provide reliable measurements in children.

## Background

The Southampton Hand Assessment Procedure (SHAP) is a measurement instrument of the functionality of normal, impaired, and prosthetic hands [1]. Currently, clinicians and researchers prefer to use the SHAP [2-9] because it provides a comprehensive overview of the functionality of prehensile grips (spherical, tripod, power, lateral, tip, and extension) and a general functionality score. SHAP scores are calculated based on the execution times of its tasks. SHAP tasks are designed to evaluate unilateral hand function of adults [1]. Although assessing hand function is equally important for the adult as the pediatric population, currently no version of the SHAP exists for children.

Several instruments are available for evaluating hand functioning of children with different hand impairments, such as spastic hand due to cerebral palsy (CP; 20.8/10.000 births) [10], upper limb reduction deficiencies (ULRD; 5.0 births/10.000) [11], or traumatic injuries of the hand (41% of childhood injuries) [12]. For instance, the Melbourne Assessment of Unilateral Upper Limb Function (Melbourne assessment) and the Quality of Upper Extremity Skills Test (QUEST) [13-15] are used in children with different types of CP. The Assessment of Capacity for Myoelectric Control (ACMC) evaluates functioning with a prosthesis, the University of New Brunswick Test (UNB) focuses on bimanual functioning, and the Assisting Hand Assessment (AHA) evaluates the role of the impaired hand as assisting hand for the unimpaired hand [16-18]. These measurement instruments are recommended for clinical use. However, some measurement instruments require extended training of the assessor (e.g., ACMC), which may limit clinical applicability [19-21]. The instruments focusing on evaluating bilateral functioning [16,22,23] cannot assess the capabilities of the affected hand alone (e.g., UNB). Some instruments focus on evaluating the functionality of prosthetic hands (e.g., ACMC and other, see the literature reviews) [19,20,24,25] or of hands subject to the specific effects of conditions such as CP or ULRD (e.g., Melbourne assessment, AHA) [14,16]. Furthermore, the outcomes of the existing instruments lack description about the functionality of different hand grips. With high incidences and various impairments of the hand, healthcare professionals need an accurate instrument that does not require formal training and can be used for multiple hand impairments. Such a broad-applicability instrument would enable comparison of the functionality scores of different impairments of pediatric hands with regard to unimpaired hand functioning.

The SHAP is an instrument that has web-based training [26,27] and makes comparisons between scores of unimpaired, impaired, and prosthetic hands possible. The SHAP provides scores for the functionality that are calculated relative to norms [1,28]. The SHAP reliability and norm values were determined using unimpaired young adults that were considered to have optimal hand functionality [1]. Thus, SHAP functionality scores of any type of hand impairment are relative to optimal hand functionality (of unimpaired young adults). However, the SHAP has not been used in children thus far, and the relevant clinimetric properties have yet not been established in children. To use the SHAP for children (SHAP-C), several steps are required:

a. Adjustment of the objects used to perform the tasks and the SHAP protocol for a specific age group and size of the impaired/unimpaired hand or prosthesis, as some of the SHAP objects are relatively heavy and large for a child’s hand or prosthesis [16];

b. Testing of the reliability in unimpaired children and determination of the norm values for unimpaired children;

c. Testing of SHAP-C validity; and

d. Testing of the reliability in children with prosthetic hands and other hand impairments because the SHAP was originally designed to evaluate unimpaired, impaired, and prosthetic hands.

This study focused on the first steps, adjusting the SHAP, providing norm values, and testing reliability in children with unimpaired upper limbs.

The aims of the study were as follows: (1) to modify the objects and protocol of SHAP for children’s hands or prostheses, (2) to provide norm data about the means of SHAP-C tasks for 4- to 6-y/o boys and girls with unimpaired hands, and (3) to assess the inter- and intra-rater reliability of the SHAP-C in these children.

## Methods

### SHAP-C

The SHAP consists of 26 tasks: 12 tasks with abstract objects and 14 tasks concerning activities of daily living (ADL, Table 1). The time needed to complete each task is recorded in seconds. Using z-score transformations of task-times and the Euclidean distance, six prehensile patterns and a general index of function (IOF) are computed. All six scores of prehensile patterns form the functionality profile (FP). The prehensile patterns and IOF are calculated relative to the predetermined norms and represent the functionality scores of hand grips (Spherical, Tripod, Power, Lateral, Tip, Extension) [1]. The FP and IOF scores range from 1 to 100 (100 is normal functionality). Scores higher than 100 are possible if the assessed person performs better compared with the normative data. The normative data for the tasks in adults range from a mean performance time of 1.58 seconds to 1.84 seconds in abstract objects tasks and from 3.12 seconds to 6.77 seconds in ADL tasks. The normative data for the prehensile patterns and the IOF are not available in the literature because of the intellectual property rights of parties commercializing SHAP. The test-retest reliability of SHAP has been tested in unimpaired young adults using analysis of variance (ANOVA) [1]. The ANOVA F-values of SHAP tasks and the FP and IOF functionality scores do not exceed F_critical_ = 3.28, indicating there is no difference between the replicates and demonstrating, thus, good reliability [1,28].

**Table 1:** SHAP functionality profiles, tasks and modifications to the tasks for use in the SHAP-C

**SHAP**			**SHAP-C adjustments**	
**FP**	**AO tasks**	**Objects and protocol**^**a**^	**Downsized objects**	**Modified protocol**
S	Light sphere	Wooden sphere transferred from location 1 to location 2	Sphere (diam. = 3 cm, weight = 1 g)	-
Tr	Light tripod	Wooden prism transferred from location 1 to location 2	-	-
P	Light power	Wooden cylinder transferred from location 1 to location 2	Cylinder (height = 10 cm, diam. = 3 cm, weight =5 g)	-
L	Light lateral	Wooden shape in form of a square mug transferred from location 1 to location 2	-	The object is positioned with the handle lateral to the side where the participant had the simulator on, not facing the participant.
Tp	Light tip	Wooden rectangular prism transferred from location 1 to location 2	-	-
E	Light extension	Wooden rectangular prism transferred from location 1 to location 2	-	-
S	Heavy sphere	Metal sphere transferred from location 1 to location 2	Sphere (diam. = 3 cm, weight = 37 g)	-
Tr	Heavy tripod	Metal prism transferred from location 1 to location 2	-	-
P	Heavy power	Metal cylinder transferred from location 1 to location 2	Cylinder (height = 10 cm, diam. = 3 cm, weight = 187 g)	-
L	Heavy lateral	Metal shape in form of a squared mug transferred from location 1 to location 2	-	The object is positioned with the handle lateral to the side where the participant had the simulator on, not facing the participant.
Tp	Heavy tip	Metal rectangular prism transferred from location 1 to location 2	-	-
E	Heavy extension	Metal rectangular prism transferred from location 1 to location 2	-	-
	ADL tasks			
Tp	Pick up coins	Picking up independently four coins and placing them into a jar.	-	The coins were dragged to the edge of the table by the participant. The assessor held the coins with index finger at the edge of the table while the participant picked up the coin and placed it in a jar.^b^
Tr + Tp	Undo buttons	Undoing four different sized buttons.	-	-
Tr + P	Food cutting	Cutting a plasticine block with a knife.	-	The assessor helped with fixating the knife in the prosthesis.^b^
E	Page turning	Pick up a 4 × 6 inch (10.2 × 15.2 cm) card from location 1, turn it over, and place it in location 2.	-	-
S	Remove jar lid	Pick up the jar with the non-assessed hand, open the lid with the assessed hand using a flexion grip with the lid in the palm.	Smaller jam jar (diam. = 4 cm, diam. lid = 4.2 cm, height = 9 cm, weight = 76 g)	
L	Pour water from jug	Place the jug with 100 ml of water with the handle oriented to the side of the assessed hand. Lift it up by the handle and pour the water into a jar.	Only 50 ml water was used.	-
S	Pour water from carton	Fill in the carton with 200 ml of water, grasp it, and pour the water into the jar.	Smaller juice carton with 100 ml water (length = 5.1 cm, width = 3.7 cm, height = 11 cm, weight = 6 g)	-
P	Move a full jar	Lift a full jar with water from location 1 to location 2 over a barrier (the carton). Location 1 of the jar is the opposite side of the assessed hand.	Smaller jam jar 150 ml water (diam. = 3.7 cm, height 10.1 cm, weight = 91 g)	-
P	Move an empty tin	Lift an empty tin from location 1 to location 2 over the barrier (the carton). Location 1 of the jar is the opposite side of the assessed hand.	Smaller tin (diam. = 3.5 cm, height = 9 cm, weight = 11 g)	-
L + E	Move a tray	The tray is placed on the opposite side of the assessed hand. The closed SHAP unit is placed with the longer side facing the participant to serve as a barrier. Using both hands and remaining seated, pick up the tray from location 1, pass it over the barrier and place it in location 2.	Lighter tray (length = 42 cm, width = 26 cm, weight = 558 g)	The unit kit was placed with the shorter side facing the participant.
L + Tp	Rotate a key 90°	Rotate the key from a vertical position 90° to a white mark using a lateral grip.	-	-
L + Tp	Open/close a zip	Open and close a zipper.	An extension to the zipper’s pull-tab (paperclip)	The assessor held the pull-tab for easier grasping.^b^
P	Rotate a screw 90°	The screwdriver is placed on the form-board on the side of the assessed hand. The screw is clipped on the exterior of SHAP unit on the side of the assessed hand. Both hands can be used to guide the screwdriver to the screw, but only the assessed hand is turning the screwdriver.	-	The participant picked up the screwdriver with the non-assessed hand and passed it over to the assessed hand. The assessor helped with fixating the screwdriver in the prosthesis.^b^
P	Rotate a door handle	With the assessed hand, rotate a door handle until open.	-	-

Modifications in SHAP-C: (1) downsized objects, (2) slightly modified positioning of a few objects on the form-board, and (3) the assessor timed the tasks and not the participant.

*Abbreviations and notations*: SHAP–Southampton Hand Assessment Procedure, SHAP-C–Southampton Hand Assessment Procedure for Children, AO–abstract objects, ADL–activities of daily living, FP–functionality profile, S–Spherical (n = 4 tasks), Tr–Tripod (n = 4 tasks), P–Power (n = 7 tasks), L–Lateral (n = 6 tasks), Tp–Tip (n = 6 tasks), E–Extension (n = 4 tasks), ‘-’ no modification was applied.

^a^The tasks are executed with the hand under assessment. Location 1 and location 2 are specified on the form-board for each task.

^b^Adjustments performed for the prosthetic hands (based on the pilot study); for non-disabled hands, the adjustment was unnecessary.

**Figure 1:**
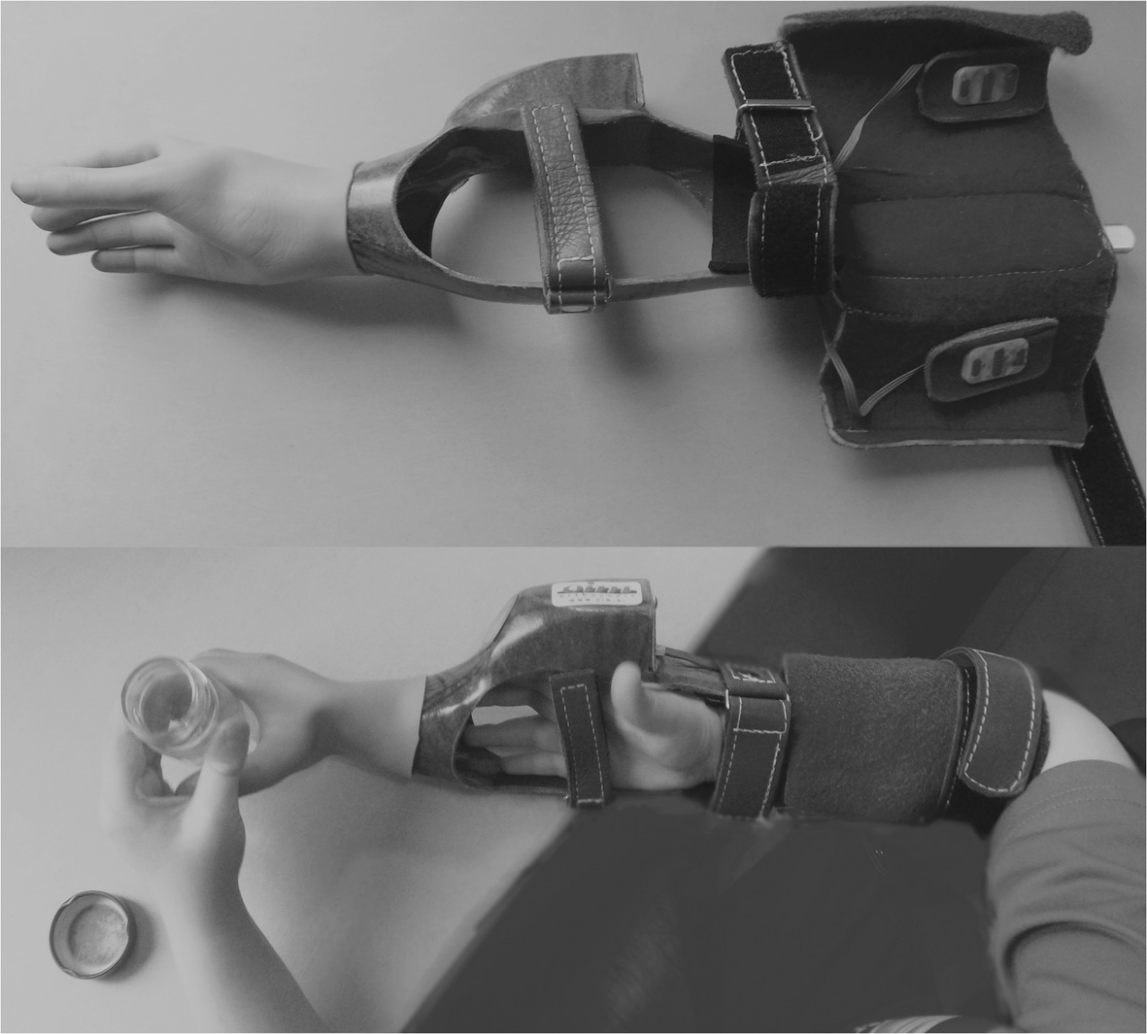
Prosthetic simulator.

In the process of establishing the SHAP-C, we focused on keeping the alterations of the original SHAP to a minimum. Therefore, a systematic approach was used for designing the SHAP-C. (1) First, several objects were downsized (Table 1) to allow grasping with both pediatric unimpaired and prosthetic hands, as the SHAP was designed for prosthetic hands as well (maximum opening of the prosthetic hand, distance from thumb to index finger is 5 cm [myoelectric prosthesis, Electrohand 2000]). Object sizes and the original SHAP protocol [27] were tested in a pilot study on eight unimpaired children (4–7 y/o, three girls and five boys). The children were recruited from a local school. They performed with a normal hand or with a myoelectric prosthesis adapted for the use in unimpaired children (a prosthetic simulator, Figure 1). (2) After that pilot study, it was decided that the assessor will time the tasks instead of the child as stated in the standard SHAP protocol because the children often forgot to start and stop the timer. Timing was started at the moment of opening the hand to grasp the object and stopped when the object was released. Furthermore, all of the objects (including the resized objects) and the changed SHAP protocol were tested in three other children (5 y/o), using the myoelectric simulator, to evaluate the feasibility of the SHAP-C protocol in pediatric prosthetic hands as well. (3) Based on the observations from the children using the prosthetic simulator, the starting position of a few objects was slightly changed to facilitate the gripping of the objects in prosthesis users (Table 1).

### Participants’ norm values and reliability study

The children were recruited from two local primary schools on a voluntary basis. Children were unimpaired, right-handed and were included if they were four to six years old (y/o) (primary school starts at 4 y/o in the Netherlands), free of upper limb musculoskeletal or neurologic disorders, had normal/corrected to normal sight and were not familiar with the SHAP. As we would like the SHAP-C also to be available for pediatric prosthesis users in the future, we included 4-to 6-y/o children. This age group was defined according to the size (opening width) and functional abilities of a generally used prosthesis hand (Otto Bock, Electrohand 2000), appropriateness of the SHAP-C tasks in children, and ability to receive and follow tasks instructions.

Study approval was granted by the Medical Ethical Committee (NL35268.042.11). A parent (or guardian) provided written informed consent and filled in a short questionnaire about age, gender, and hand dominance of their child. All of the children received a gift toy (value ± 5 Euro) at the completion of the measurements.

#### Procedure

A repeated-measures study was set up to evaluate intra- and inter-rater reliability of the SHAP-C. The children were assigned to perform the tasks every session with the same hand, dominant (right hand) or non-dominant hand. Dominant and non-dominant hand performance was needed to obtain a better representation of the tasks means. Measurements were performed in a quiet classroom at the primary school. The child and two assessors were present. First, the child was seated comfortably on a chair, and, when needed, height was adjusted to allow 90° elbow flexion when the hand rested on the table. Each SHAP-C task was first demonstrated by the assessor. The tasks had to be executed as accurate and as fast as possible. Children started to open the hand when near the object. For each object, a start position and an end position were specified with molds on a board (form-board) that was lying on the table in front of the child. Before executing the abstract objects tasks, the corresponding mold on the form-board was aligned to meet the middle line of the participant to standardize testing in both conditions with the dominant or non-dominant hand. No prior practice was allowed and repetition of a task was performed when the child did not complete the task according to the exact requirements (appropriate grip, object location) [27].

Three assessors collected the measurements. The assessors were instructed by a detailed SHAP protocol, which was accompanied by videos demonstrating the tasks and time measurement [26,27]. They read and understood the modifications of the SHAP-C and practiced SHAP-C during the pilot study. Assessor 1 and assessor 3 had previous experience in applying the SHAP, which was not the case for assessor 2. A verbal signal to start the task was given to the child. The tasks were executed in random order to avoid any sequence effects. In total, four measurement sessions of the SHAP-C were collected during four consecutive days. Children participated in one SHAP-C session per day with approximately 24 h between sessions. The SHAP-C results of the four sessions were used to determine the norm values.

#### Intra- and inter-rater reliability

Assessor 1 measured the task times of two SHAP-C sessions (day 1 and day 2). Assessor 2 measured the times of one SHAP-C session on day 3. Assessor 3 measured the times of one SHAP-C session on day 4.

### Statistical analysis

In this study, the task times, denoting performance times in each SHAP-C task, were used for the analyses.

#### Norm values

First, the task means of the four sessions were calculated per participant. Second, to determine the norm values, the means and the standard deviations of each SHAP-C task were calculated based on the means of the four sessions. Independent samples t-tests were used to determine differences between boys and girls. The test results of the ‘equal variances not assumed’ row were reported when the homogeneity of variances assumption was violated. In addition, we tested the differences between performance with dominant and non-dominant hands with a t-test.

#### Intra-rater reliability

The paired samples t-test was used to analyze the differences between the task times of the first and second session of assessor 1. A repeatability coefficient (RC) was determined for each SHAP-C task [29,30]. The RC is defined as the value in which the differences between repeated measurements are expected to lie with a 95% probability and is calculated as 1.96 × s ×  (s, within-subject standard deviation) [29,30]. The relative RCs, the percentage of variance of the RC outcome from the mean, were also calculated and constituted the primary outcome measure.

#### Inter-rater reliability

Repeated-measures ANOVA was used to analyze the differences between the task times of the second SHAP-C session (assessor 1), the third session (assessor 2), and the fourth session (assessor 3). When sphericity was violated, Greenhouse-Geisser correction for the degrees of freedom was applied. Bonferroni correction was applied for the post-hoc test. For each SHAP-C task, the agreement between the assessors was determined by calculating the RC and the relative RC.

We considered values of ≤ 75% for relative RC as clinically acceptable values for variation of task times from the mean denoting acceptable reliability. Statistical significance for analyses was P ≤ 0.05 (two-sided) and analyses were performed using SPSS Statistics for Windows, version 20.0 (IBM Corp., 2011, Armonk, NY, http://www.spss.com).

## Results

In total, 24 children participated, and 54% were boys. The mean age was 5 y/o [SD = 0.54], and the dominant: non-dominant hand ratio was 8:5 for boys and 5:6 for girls.

### SHAP-C feasibility and task means

All children were able to grip the resized objects with their hand. The means for the abstract objects varied between 0.75 and 1.21 seconds, and the means for ADL tasks varied per task, with the highest mean of 19.13 seconds for the undo buttons task (Table 2). Girls were slower than boys in five SHAP-C tasks: light extension (P = 0.006), heavy lateral (P = 0.012), heavy extension (P = 0.018), pour water from jug (P = 0.044) and open/close a zipper (P = 0.007, Table 2). Participants performing with the dominant hand were faster in the heavy extension, food cutting and page turning tasks (P-values < 0.01).

**Table 2:** The norm values of SHAP-C tasks in unimpaired children

**SHAP-C tasks**	**Total (n = 24)**	**Girls (n = 11)**	**Boys (n = 13)**			**Non-dominant hand (n = 11)**	**Dominant hand (n = 13)**		
	**Mean [SD] (seconds)**	**Mean [SD] (seconds)**	**Mean [SD] (seconds)**	**Difference in means [SE] (seconds)**	**P**	**Mean [SD] (seconds)**	**Mean [SD] (seconds)**	**Difference in means [SE] (seconds)**	**P**
AO tasks									
Light sphere	0.75 [0.18]	0.81 [0.21]	0.71 [0.15]	0.10 [0.07]	0.203	0.79 [0.11]	0.73 [0.22]	0.06 [0.07]	0.418
Light tripod	0.78 [0.15]	0.83 [0.14]	0.74 [0.15]	0.09 [0.06]	0.123	0.82 [0.13]	0.76 [0.16]	0.06 [0.06]	0.327
Light power	0.77 [0.21]	0.83 [0.21]	0.72 [0.21]	0.10 [0.09]	0.248	0.77 [0.14]	0.77 [0.26]	−0.003 [0.08]	0.976^a^
Light lateral	0.90 [0.16]	0.93 [0.17]	0.88 [0.16]	0.05 [0.07]	0.434	0.90 [0.13]	0.90 [0.19]	−0.01 [0.07]	0.910
Light tip	0.78 [0.13]	0.81 [0.12]	0.75 [0.13]	0.06 [0.05]	0.236	0.78 [0.12]	0.77 [0.13]	0.01 [0.05]	0.897
Light extension	0.87 [0.21]	1.00 [0.22]	0.77 [0.15]	0.23 [0.07]	0.006*	0.93 [0.21]	0.82 [0.21]	0.11 [0.09]	0.230
Heavy sphere	0.79 [0.15]	0.84 [0.19]	0.75 [0.11]	0.09 [0.06]	0.157	0.79 [0.12]	0.80 [0.18]	−0.002 [0.06]	0.970
Heavy tripod	0.84 [0.23]	0.88 [0.17]	0.81 [0.27]	0.08 [0.09]	0.435	0.89 [0.20]	0.80 [0.25]	0.09 [0.09]	0.370
Heavy power	0.83 [0.22]	0.90 [0.26]	0.77 [0.16]	0.13 [0.09]	0.155	0.84 [0.24]	0.82 [0.22]	0.02 [0.09]	0.856
Heavy lateral	1.21 [0.31]	1.37 [0.23]	1.07 [0.30]	0.31 [0.11]	0.012*	1.19 [0.27]	1.22 [0.35]	−0.02 [0.13]	0.869
Heavy tip	0.95 [0.17]	1.00 [0.17]	0.91 [0.15]	0.10 [0.07]	0.161	0.99 [0.21]	0.92 [0.12]	0.07 [0.07]	0.325a
Heavy extension	0.95 [0.28]	1.10 [0.32]	0.82 [0.16]	0.28 [0.11]	0.018^a^*	1.12 [0.29]	0.80 [0.17]	0.32 [0.10]	0.006^a^*
ADL tasks									
Pick up coins	5.64 [1.03]	5.92 [1.15]	5.40 [0.89]	0.52 [0.42]	0.221	5.92 [1.07]	5.40 [0.97]	0.52 [0.42]	0.221
Undo buttons	19.13 [6.57]	16.51 [5.27]	21.35 [6.93]	−4.83 [2.55]	0.072	17.95 [7.67]	20.13 [5.60]	−2.18 [2.71]	0.430
Food cutting	5.74 [1.61]	6.28 [1.79]	5.29 [1.34]	0.99 [0.64]	0.137	6.87 [1.69]	4.78 [0.64]	2.09 [0.54]	0.002^a^*
Page turning	1.37 [0.55]	1.55 [0.56]	1.21 [0.52]	0.33 [0.22]	0.144	1.64 [0.49]	1.14 [0.52]	0.50 [0.21]	0.025*
Remove jar lid	2.19 [0.95]	2.44 [1.00]	1.98 [0.90]	0.45 [0.39]	0.253	2.43 [0.99]	1.98 [0.90]	0.45 [0.39]	0.261
Pour water from jug	5.12 [1.51]	5.84 [1.78]	4.52 [0.91]	1.31 [0.59]	0.044^a^*	5.52 [1.75]	4.79 [1.23]	0.73 [0.61]	0.243
Pour water from carton	9.09 [2.56]	9.73 [2.94]	8.55 [2.16]	1.18 [1.04]	0.271	8.84 [2.55]	9.31 [2.65]	−0.47 [1.07]	0.665
Move a full jar	0.96 [0.25]	1.02 [0.28]	0.91 [0.23]	0.11 [0.10]	0.296	1.03 [0.27]	0.90 [0.24]	0.13 [0.10]	0.225
Move an empty tin	0.84 [0.22]	0.89 [0.17]	0.79 [0.25]	0.11 [0.09]	0.239	0.89 [0.18]	0.79 [0.25]	0.10 [0.09]	0.285
Move a tray	3.15 [1.13]	3.63 [1.09]	2.74 [1.03]	0.89 [0.44]	0.053	3.22 [1.17]	3.09 [1.15]	0.13 [0.47]	0.794
Rotate a key 90°	0.76 [0.14]	0.77 [0.16]	0.76 [0.14]	0.01 [0.06]	0.810	0.73 [0.10]	0.79 [0.17]	−0.06 [0.06]	0.297
Open/close a zip	2.09 [0.70]	2.49 [0.69]	1.75 [0.52]	0.74 [0.25]	0.007*	1.95 [0.72]	2.22 [0.68]	−0.27 [0.29]	0.357
Rotate a screw 90°	7.04 [1.73]	7.31 [1.95]	6.81 [1.56]	0.50 [0.72]	0.494	7.51 [2.02]	6.64 [1.40]	0.87 [0.70]	0.227
Rotate a door handle	0.64 [0.12]	0.66 [0.14]	0.63 [0.12]	0.03 [0.05]	0.555	0.67 [0.11]	0.61 [0.13]	0.06 [0.05]	0.273

*Abbreviations and notations*: SHAP-C–Southampton Hand Assessment Procedure for Children, AO–abstract objects, ADL–activities of daily living, SD–standard deviation, SE–standard error, P–significance value.

^a^P-values for equal variances not assumed (homogeneity assumption of t-test analysis was violated).

*Significance P < 0.05.

### Intra-rater reliability

#### Abstract objects

The mean task times of assessor 1 in session 2 were significantly lower than the times in session 1 for light lateral (P = 0.044) and for heavy power (P = 0.049; Table 3).

**Table 3:** Inter- and intra-rater reliability

**SHAP-C tasks**	**Assessor 1**	**Assessor 1**	**Assessor 2**	**Assessor 3**	**Intra-rater reliability: Assessor 1**				**Inter-rater reliability**			
	**Session 1**	**Session 2**	**Session 3**	**Session 3**	**(Session 1 and session 2)**				**Assessor 1 (Session 2), Assessor 2 and Assessor 3**			
	**Mean [SD] (seconds)**	**Mean [SD] (seconds)**	**Mean [SD] (seconds)**	**Mean [SD] (seconds)**	**Mean difference [SD] (seconds)**^**a**^	**P**^**a**^	**RC (seconds)**	**RRC (%)**	**F-ratio**^**b**^	**P**^**b**^	**RC**^**b **^**(seconds)**	**RRC (%)**
AO tasks												
Light sphere	0.77 [0.19]	0.70 [0.17]	0.90 [0.53]	0.65 [0.25]	0.07 [0.28]	0.221	0.55	74.4	F(1.3, 29.2) = 3.60	0.059	0.98	130.6
Light tripod	0.84 [0.20]	0.83 [0.21]	0.75 [0.24]	0.71 [0.35]	0.01 [0.26]	0.927	0.51	60.6	F(2, 46) = 1.39	0.259	0.70	91.6
Light power	0.88 [0.33]	0.78 [0.27]	0.83 [0.52]	0.60 [0.26]	0.10 [0.34]	0.144	0.68	82.3	F(2, 46) = 2.93	0.063	0.98	133.8
Light lateral	0.98 [0.25]	0.87 [0.20]	0.84 [0.30]	0.93 [0.34]	0.11 [0.25]	0.044*	0.53	58.0	F(2, 46) = 0.68	0.513	0.77	88.1
Light tip	0.80 [0.22]	0.78 [0.27]	0.78 [0.30]	0.75 [0.30]	0.02 [0.36]	0.790	0.68	86.6	F(2, 46) = 0.10	0.904	0.80	103.3
Light extension	0.96 [0.40]	0.89 [0.23]	0.66 [0.24]	0.98 [0.46]	0.07 [0.37]	0.365	0.72	77.5	F(2, 46) = 6.88	0.002*	0.93	110.2
Heavy sphere	0.83 [0.24]	0.80 [0.16]	0.76 [0.27]	0.78 [0.26]	0.03 [0.25]	0.521	0.49	59.3	F(2, 46) = 0.26	0.775	0.58	74.3
Heavy tripod	0.88 [0.29]	0.87 [0.37]	0.74 [0.28]	0.88 [0.46]	0.01 [0.48]	0.906	0.92	105.1	F(2, 46) = 1.42	0.252	0.91	109.7
Heavy power	0.95 [0.38]	0.80 [0.15]	0.76 [0.39]	0.79 [0.51]	0.14 [0.34]	0.049*	0.71	81.5	F(2, 46) = 0.10	0.901	0.97	123.3
Heavy lateral	1.24 [0.38]	1.22 [0.43]	1.23 [0.49]	1.13 [0.46]	0.03 [0.29]	0.663	0.56	45.6	F(2, 46) = 0.48	0.623	1.07	89.7
Heavy tip	1.03 [0.28]	1.01 [0.31]	0.79 [0.35]	0.97 [0.48]	0.01 [0.35]	0.845	0.67	65.9	F(1.7, 38.0) = 1.89	0.171	1.20	130.0
Heavy extension	0.96 [0.32]	0.91 [0.24]	0.87 [0.28]	1.05 [0.57]	0.05 [0.30]	0.389	0.58	62.5	F(1.4, 32.8) = 2.24	0.136	0.88	93.8
ADL tasks												
Pick up coins	6.28 [2.07]	5.71 [1.28]	5.14 [0.93]	5.42 [1.16]	0.57 [1.99]	0.173	3.98	66.3	F(2, 46) = 2.64	0.082	2.49	46.0
Undo buttons	22.76 [10.84]	20.91 [9.83]	18.60 [12.35]	14.17 [6.58]	1.84 [9.65]	0.359	18.87	86.4	F(1.6, 34.9) = 3.70	0.044*	24.09	134.7
Food cutting	7.08 [2.89]	5.93 [1.53]	4.41 [2.20]	5.54 [2.09]	1.14 [2.29]	0.023*	4.94	75.9	F(2, 46) = 6.05	0.005*	4.80	90.7
Page turning	1.75 [0.81]	1.41 [0.55]	1.26 [0.71]	1.06 [0.51]	0.33 [0.52]	<0.01*	1.18	75.0	F(2, 46) = 5.40	0.008*	1.14	92.0
Remove jar lid	2.44 [1.09]	2.20 [1.01]	2.22 [1.52]	1.89 [1.15]	0.24 [0.65]	0.080	1.33	57.2	F(1.7, 39.4) = 1.02	0.359	2.47	117.2
Pour water from jug	6.08 [2.22]	5.08 [1.27]	4.75 [2.27]	4.59 [1.62]	1.00 [1.71]	<0.01*	3.82	68.5	F(2, 46) = 1.01	0.371	3.34	69.5
Pour water from carton	10.85 [3.80]	8.88 [2.13]	8.59 [3.16]	8.06 [3.62]	1.97 [3.22]	<0.01*	7.29	73.9	F(2, 46) = 1.03	0.366	5.59	65.7
Move a full jar	1.02 [0.36]	1.00 [0.29]	0.75 [0.24]	1.05 [0.58]	0.02 [0.32]	0.732	0.61	60.5	F(1.4, 32.0) = 5.09	0.021*	1.02	109.5
Move an empty tin	0.91 [0.31]	0.84 [0.27]	0.87 [0.60]	0.74 [0.27]	0.07 [0.30]	0.245	0.58	67.1	F(1.7, 38.3) = 0.69	0.481	1.10	135.3
Move a tray	3.00 [1.23]	2.98 [1.16]	3.58 [1.32]	3.05 [1.52]	0.02 [0.91]	0.903	1.74	58.2	F(2, 46) = 3.95	0.026*	2.40	75.0
Rotate a key 90°	0.75 [0.35]	0.70 [0.12]	0.72 [0.25]	0.88 [0.40]	0.04 [0.31]	0.492	0.60	82.9	F(1.7, 38.3) = 2.82	0.081	0.83	107.8
Open/close a zip	2.06 [0.98]	2.15 [1.04]	2.12 [0.88]	2.04 [1.10]	−0.09 [1.00]	0.649	1.93	91.4	F(2, 46) = 0.11	0.896	2.37	112.7
Rotate a screw 90°	7.98 [2.86]	7.47 [2.06]	5.55 [2.40]	7.17 [2.50]	0.51 [2.67]	0.359	5.22	67.6	F(2, 46) = 6.60	0.003*	6.07	90.3
Rotate a door handle	0.70 [0.21]	0.60 [0.13]	0.57 [0.22]	0.70 [0.29]	0.10 [0.21]	0.030*	0.45	70.1	F(2, 46) = 2.58	0.087	0.58	93.7

*Abbreviations and notations*: SHAP-C–Southampton Hand Assessment Procedure for Children, AO–abstract objects, S–session, A–assessor, SD–standard deviation, RC–repeatability coefficient, RRC–relative repeatability coefficient, P–significance value.

^a^Paired t-test for the results of the first and the second session of assessor 1.

^b^Repeated measures ANOVA for the results of assessor 1 (second session) assessor 2 and assessor 3.

*Significance P < 0.05.

Tasks RCs varied from 0.51 to 0.92 seconds in the abstract objects tasks. Relative to the mean of the first two sessions, values ≤ 75% were observed for the relative RCs in 7/12 abstract objects tasks. Light power, light tip, light extension, heavy tripod, and heavy power displayed relative RCs > 75%.

#### ADL tasks

The t-test indicated significantly lower means in session 2 compared with session 1 in the tasks: food cutting (P = 0.023), page turning (P < 0.01), pouring water from jug (P < 0.01), pouring water from carton (P < 0.01), and rotating a door handle (P = 0.030, Table 3).

The RCs of 10 out of the 14 ADL tasks were lower than 75% from the tasks means. In the undo buttons, food cutting, rotate a key 90°, and open/close a zipper task, the relative RCs exceeded 75% from the task mean (Table 3).

### Inter-rater reliability

#### Abstract objects tasks

No significant differences in the time means of the three assessors were found, except for the light extension task (P = 0.002) (Table 3). In the post hoc analysis, the mean of assessor 2 was significantly lower than those of assessor 1 and assessor 3 (P = 0.016 and P = 0.010, respectively).

The RC values ranged from 0.58 to 1.20 seconds in the abstract object tasks (Table 3). The relative RC values between the three raters were all > 75%, except for the heavy sphere task (relative RC = 74.3%).

#### ADL tasks

The assessors differed significantly in the task times in 6 out of 14 ADL tasks: undo buttons (P = 0.044), food cutting (P = 0.005), page turning (P = 0.008), move a full jar (P = 0.021), move a tray (P = 0.026), and rotate a screw 90° (P = 0.003) (Table 3). Post hoc analyses revealed that assessor 2 recorded lower means than both assessor 1 (P = 0.006) and assessor 3 (P = 0.038) for the rotating a screw 90°, than assessor 1 in food cutting (P = 0.014) and in moving a full jar (P = 0.001) and differed from assessor 3 in moving a tray (P = 0.054, mean_assessor2_ > mean_assessor3_). Between assessor 1 and assessor 3, significant differences were observed in undoing buttons (P = 0.034) and in page turning (P = 0.003); assessor 3 recorded lower means.

In all ADLs, task times varied within the RC ≤ 6.07 seconds, except for the undo buttons (task in which RC = 24.1 seconds). The relative RCs were > 75% in the majority of ADLs. For four tasks, the relative RCs were ≤ 75%: pick up coins, pour water from jug, pour water from carton, and move a tray.

## Discussion

This is the first study to adapt the SHAP for pediatric use and assessed reliability of this adapted version, the SHAP-C. Children were able to perform all SHAP-C tasks using the corresponding objects (including the downsized objects). The task means were significantly different in 7/26 tasks when a single assessor tested twice and in 7/26 tasks when three different assessors tested (P-values < 0.05). The intra-rater reliability of the SHAP-C was relatively better compared with the inter-rater reliability. Variation values within the same assessor, the RCs, had percentages < 75% in 17 out of 26 SHAP-C tasks (7/12 abstract objects tasks and 10/14 ADL tasks), indicating a relatively good repeatability of the procedure within the same assessor, at least in ADL tasks. The time scores per task varied largely between the three assessors. In 22/26 SHAP-C tasks, the RCs were higher than 75% of the task mean, thus revealing, poor SHAP-C repeatability. The small differences in task means on a group level indicate that the SHAP-C can be used for group comparisons. However, in clinical practice on an individual level, the SHAP-C may be used when one assessor is engaged but with considerable within-subject variation (Table 3). The SHAP-C should be used with caution when more assessors are engaged. Further adjustments are required to provide clinicians with a reliable SHAP-C.

In the current study, the mean values for the abstract objects of the SHAP-C tasks are much lower than the means of the SHAP tasks in adults (overall means_SHAP-C_ < 1.21 seconds vs. means_SHAP_ > 1.58 seconds) [28]. This discrepancy is most likely because timing of the SHAP-C tasks was differently executed than in the SHAP (timing by the assessor vs. self-timing). Compared with the SHAP, the SHAP-C means do not include the times of two phases: (1) stopwatch activation–reaching-the-object and (2) after-release of the object–stopping the stopwatch. On the other hand, the SHAP-C means in more complex ADL tasks were overall higher than those of SHAP in adults (e.g., pick up coins, undo buttons, or food cutting, means_SHAP-C_ = 5.64-19.13 seconds vs. means_SHAP_ = 3.12-6.77 seconds) [28]. This finding of children being slower than adults in executing complex tasks is in line with the reports in literature explaining age-related differences in (neuro) motor development (e.g., maturation of neural cortex gradually over time) [31,32]. Nevertheless, our means for the SHAP-C tasks represent the first estimations of norm values. Because of the observed variability in task times (Tables 2 and 3), a larger sample is required to determine the norms once the SHAP-C protocol is more definitive.

Bland and Altman recommended the use of RC to determine consistency in outcomes of a measurement instrument [29,33]. The precision of an RC over the Pearson’s correlation coefficient and intra-class correlation has been highlighted [29,34]. There are, however, no standardized rules for interpretation of RCs. The suggested approach is that the lower the RCs are, the better the repeatability of the instrument is. The comparison of the RC to the minimum clinically important difference/change (MCID) would indicate good reliability of the instrument if the RC < MCID and vice-versa [34]. In the absence of MCID values for the SHAP or SHAP-C, we chose to represent the RCs in percentages relative to the task means as used by others [35,36]. The relative RCs quantify the degree of agreement between different or single assessors and facilitate the interpretation of RCs. The cut-off point for the relative RC (75%) was chosen arbitrarily; higher (80%) and lower (50%) cut-off points have been reported previously [35,36]. Thus, one may shift the cut-off value and interpret the RCs found in this study accordingly. Using a cut-off point of 80% for our relative RCs, for example, would have not changed the current results because all non-reliable tasks had relative RCs higher than 80%.

### Intra-rater reliability

The majority of tasks were reliable (relative RCs < 75% in 17/26 tasks) for assessor 1. In comparison with the adult version of the SHAP intra-rater reliability, we found approximately the same amount of less replicable tasks. In the SHAP, seven tasks have been found to be less reliable (light power, light tip, heavy extension, page turning, pour water from carton, rotate a key 90°, rotate a screw 90°), but not to a significant extent [28]. In the SHAP-C, nine tasks were significantly less reliable (light power, light tip, light extension, heavy tripod, heavy power, undo buttons, food cutting, rotate a key 90°, and open/close a zip). The difference in less-reliable tasks between adults and children may be due to age differences in motor abilities with the upper limb [31,32]. For instance, rotating a screw 90° requires fine-motor skills. In adults, the hand motor skills have been acquired to a different extent, thus the variability when rotating a screw, whereas the tested children did not vary in this task.

Interestingly, five of the SHAP-C tasks with relative RCs > 75% were abstract object tasks. In the context of SHAP-C tasks being timed by the assessor, a possible explanation might be the variation in the assessor’s reaction time, especially in rapidly executed tasks involving abstract objects (< 1.2 seconds). The literature reports a response time of 0.18-0.20 seconds after visual stimuli and that many factors account for reaction response: practice, gender, age, fatigue, distraction, and even breathing cycle [37]. Practice might have had a role. The first measurement of the same assessor most likely served as practice and led to lower scores (faster performance) in the second measurement. We cannot exclude the fact that learning effects of SHAP-C tasks within a child might have occurred, but distinguishing learning effects from the reaction time of the assessor is not possible in this case.

An alternative, more objective method for SHAP-C data collection would be to use a different timing system. Possibly, a system that recognizes a certain opening of the thumb-index finger angle or the lifting of the hand from the table, in combination with sensors able to detect movement of position of the objects, would time the performance more accurately. Solutions for recording performance accurately can be extended to computerized systems able to depict the hand positions and objects’ shapes [38]. The inclusion of kinematic measurements would provide information about the movement time and quality of the movement. Each abstract object task may also be executed repeatedly in a certain amount of time (e.g., 10 seconds) and the number of execution times rated accordingly as in the pegboard hand-dexterity test, for example [39]. However, the mentioned solutions would increase the assessment time, costs, and dimensions of the SHAP-C kit, which is beyond the SHAP/SHAP-C purpose. With the disadvantage of increasing the time needed to determine the functionality scores, the simplest and most inexpensive approach would be to videotape the performance. Afterward, the task times could be accurately evaluated from the recordings, as has been previously accomplished for pediatric functionality tests [22,23]. Although we avoided introduction of procedures that increase data-collection or analysis time, our results suggest that these types of changes might be necessary after all, as the influence of the assessor would be diminished considerably.

### Inter-rater reliability

Clinically, the RCs of 0.58-1.20 seconds observed in abstract object tasks would be a negligible variation, but relative to the task means, this variation was rather large (≥ 75%). In this case, again, the reaction time might have had an influence on the abstract object task times [37]. Moreover, the practice experience was different across our assessors. Two of the assessors had extended experience with applying SHAP. Assessor 2, on the other hand, had no previous experience and taking into account the findings–the means of assessor 2 differed in several tasks (Table 3)–the assessors may require a longer training period prior to applying the SHAP-C. The training might include studying instructional movies centralized on an online database as in the case of the SHAP [26]. Creating a benchmark test to evaluate assessors’ instructional and data-collection skills after the training would ensure a level of proficiency when applying the SHAP-C.

Furthermore, distraction, another risk-factor for variation in performance [37], might have affected our participants. Engaging 5-y/o children in performing tasks requires good motivational techniques. Our assessors used intrinsic motivation by stimulating a playful atmosphere [40] and extrinsic motivation by rewarding performance with positive reinforcement, candy, allowing the child to color, draw or offering an animal sticker. However, the motivation of some children varied during different tasks and sessions, especially in ADLs, causing delays in performance, and thus the variability in task times. One study referred to SHAP tasks as being unattractive for children [16]. If this is the case, then substituting current SHAP-C tasks with tasks simulating activities of child play [16] and using colorful objects may improve motivation and reduce distraction. Furthermore, the necessity of providing clear instructions and using good motivational techniques in children has been emphasized in the literature about other measurement instruments for pediatric hand functionality [41]. The flow theory provides some suggestions on how to stimulate intrinsic motivation in children: use age-appropriate tasks, promote a ‘fun’ environment, provide the possibility for the children to control some of the tasks (e.g., allowing them to choose an object/task that they want to continue with), set clear and achievable goals for the tasks, and avoid negative feedback (oral or non-verbal) [42].

The observed variability of the SHAP-C means may be partly explained by the variability in (neuro) motor development in preschool children up to adolescence [43-45]. Therefore, the scores on functionality assessments have to be interpreted bearing in mind this variability in children [46]. Importantly, the timed performances in children require a standardized test, well-trained assessors, and norms for different age categories [47].

Summarizing the steps to be considered for improving reliability of SHAP-C, future research has to identify an appropriate a data-collection method that will diminish the assessor’s influence. In addition, researchers have to consider providing information to the assessors about techniques to improve motivation of children, either in the form of training or including motivational techniques in the instructions for the SHAP-C protocol.

A limitation of this study would be the relatively small sample size. Although the SHAP reliability was also determined with 24 participants [1], we feel that for pediatric populations, a larger sample size is necessary to determine the SHAP-C norms and reliability. The study design for the inter-rater reliability is limited by the fact that data were collected on separate days. A more adequate design would involve simultaneously timing of the performance of the participant by the three assessors, but the task instructions being performed by one assessor would result in potential bias for the measurements of the other two assessors. In addition, having three assessors in the same room would be overwhelming for a child. Another alternative would be to measure the participants on the same day, three times with different assessors. However, this approach was not possible because of the limited availability of the children during school hours. More importantly, fatigue and disinterest may occur if children are requested to repeat 26 tasks three times in one day. Videotaping the performance might solve this issue of three consecutive sessions and limit measurements to one-time session. In addition, the order of assessors was the same per participant and measurement day. Therefore, the task means might have been affected by the order of the assessors and/or by the measurement day. For practicality reasons, we could not randomize assessors per measurement day, but further studies should consider randomizing the assessors.

A future approach for assessing inter-rater reliability of the SHAP-C in children may consider the following: (1) allowing each child to perform the SHAP-C once with a randomly assigned assessor and (2) live broadcasting the performance of the participants to the other assessors that will measure simultaneously the performance. This way, the children will not be solicited more than once and by more than one assessor, and the possible bias of rating performance of the participants that received instructions from another assessor will be evenly distributed throughout the data.

Another limitation of this study is the inability to estimate the norms for the prehensile patterns of FP and IOF that are of interest to clinicians. Based on the means and standard deviations in our study, the estimates of norms for prehensile patterns of FP and the IOF could have been calculated, but the formulas for such calculations [1,26,28,48] are not clear for us nor to our statistician. Not having the exact procedure for determining the norm values that are needed for the calculation of FP z-scores and IOF z-scores made the calculation of FP and IOF impossible for the SHAP-C data. Explanations regarding the formulas were denied to us because of the holders’ exclusive rights on the SHAP (intellectual property).

The sizes of the objects were not systematically evaluated. Therefore, research is also needed to determine the appropriate size of the objects for the hands of older children (> 6 y/o), for larger prosthetic hands with an opening width > 5 cm or for spastic hands with an opening < 5 cm. In addition, clinimetric properties should be studied in older children because of changes in performance with age [49]. The reliability of the SHAP-C should be evaluated in different impaired hands and in prosthetic users because the SHAP was also designed for use with such patients [1]. The evaluation of learning effects of the SHAP-C in prosthetic users would be valuable for clinicians repeatedly using the SHAP-C.

## Conclusions

Adjusting SHAP objects to allow grasping with normal and prosthetic hands in 4- to 6-y/o children was performed successfully. Participants were able to perform all of the SHAP-C tasks with means from 0.64 to 19.13 seconds for the tasks. The intra-rater reliability was relatively good in comparison with the inter-rater reliability. However, more adjustments of the protocol are needed to ensure the reliability of the SHAP-C, to improve the motivation of children, to minimize the assessor influence, and to determine the norms.

## Abbreviations

SHAP: Southampton Hand Assessment Procedure; SHAP-C: Southampton Hand Assessment Procedure for Children; IOF: Index of function; FP: Functionality profile; ADL: Activities of daily living; y/o: Years old; RC: Repeatability coefficient; SD: Standard deviation.

## Competing interests

The authors declare that they have no competing interests.

## Authors’ contributions

EV, CvdS, HRM, and RB contributed to the design of the study. EV collected the data. EV, PD, and HRM contributed to the analysis of the data. EV wrote the paper. All authors have revised this manuscript for important intellectual content and have approved it for submission.

## Pre-publication history

The pre-publication history for this paper can be accessed here:

http://www.biomedcentral.com/1471-2474/15/199/prepub
